# One year follow up outcomes of endoscopic retrograde appendicitis treatment for symptomatic appendicoliths

**DOI:** 10.1016/j.isci.2026.117571

**Published:** 2026-09-24

**Authors:** Ran Li, Aihua Wang, Xiutian Zhang, Xiangming Xu, Qingqing Zhang, Tao Ji, Jiao Nie, Lin Lu, Lingjian Kong, Chao Du

**Affiliations:** 1 Department of Gastroenterology, Linyi People`s Hospital, Shandong Second Medical University, Linyi, Shandong 276300, China; 2 Chongqing Jiulongpo District Traditional Chinese Medicine Hospital, Chongqing 400039, China; 3 Department of Gastroenterology, The First Affiliated Hospital of Zhengzhou University, Zhengzhou, Henan Province 450052, China; 4 Department of Gastroenterology, Weihai Municipal Hospital, Shandong University, Weihai, Shandong 264299, China

**Keywords:** endoscopic retrograde appendicitis treatment, ERAT, symptomatic appendicolith, symptomatic relief

## Abstract

Symptomatic appendicoliths may cause recurrent abdominal pain or distension, but appendix-preserving treatment options are limited. We retrospectively evaluated endoscopic retrograde appendicitis treatment (ERAT) in 78 patients with CT- or ultrasound-confirmed appendicoliths. Symptoms were assessed using a modified abdominal pain or distension questionnaire (APQ), and outcomes were evaluated separately in adults and minors. APQ scores decreased at 1 week, 1 month, 6 months, and 1 year after ERAT. At 1 year, complete remission was achieved in 37 of 47 adults (78.7%) and 27 of 31 minors (87.1%). Multivariable Cox regression identified disease duration longer than 3 months and fecalith lumps as predictors of incomplete remission in adults, whereas NLR > 1.87 was inversely associated with this outcome. ERAT may provide sustained symptom relief while preserving the appendix, although prospective multicenter controlled studies are needed.

## Highlights

• First retrospective single-center study of ERAT for symptomatic appendicoliths (*n* = 78)
• ERAT achieved complete remission in 78.7% of adults and 87% of minors at 1 year
• Fecalith lumps/longer duration predicted incomplete remission; NLR >1.87 inversely linked

## Graphical abstract

**Figure:**
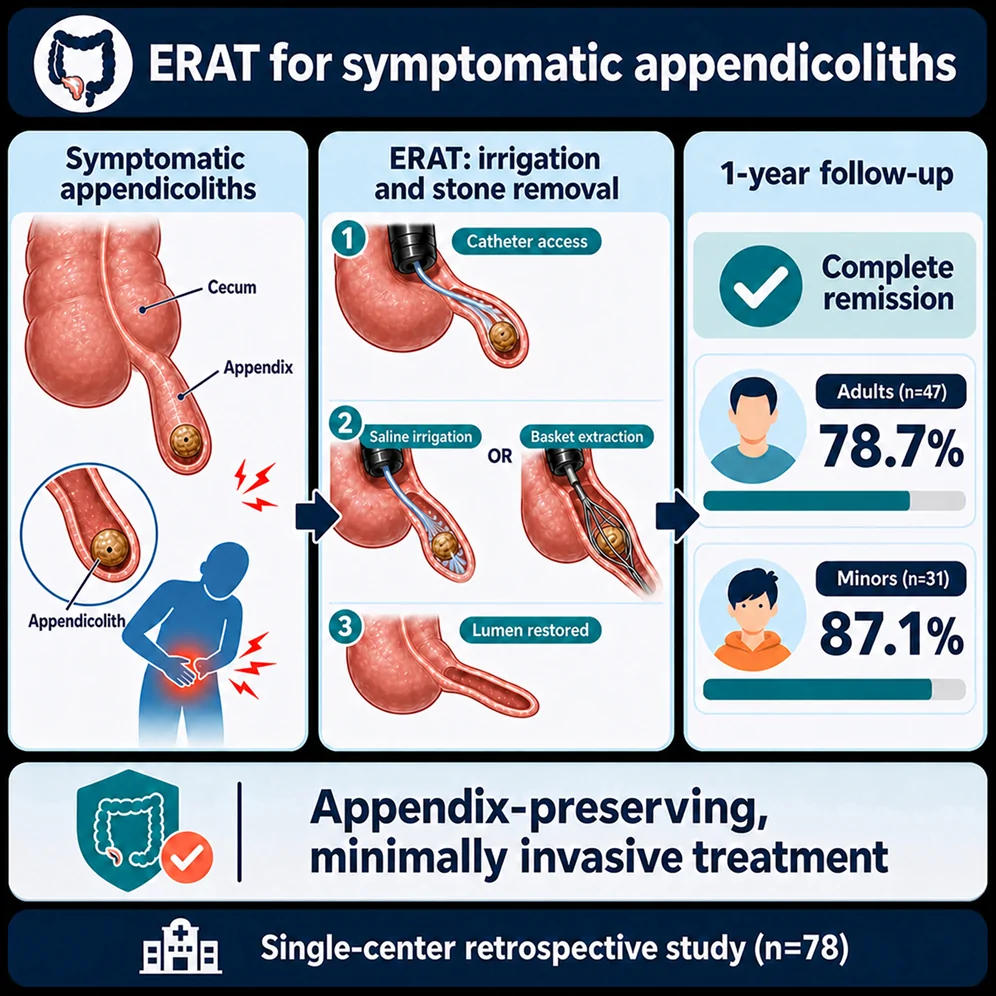

## Teaser

Health sciences; Medicine

## Introduction

Appendicolith is a calcified deposit found within the appendix, which can lead to appendicitis when it obstructs the appendiceal lumen. The presence of appendicolith is associated with a higher risk of complications and recurrence of appendicitis.^1^^,^^2^^,^^3^ It has been shown that appendicitis caused by appendicolith obstruction has a higher risk of perforation and abscess formation.^2^^,^^4^ However, appendicolith has no obvious symptoms and is often diagnosed incidentally during imaging studies performed for other reasons.^5^^,^^6^^,^^7^ As a result, the true incidence of appendicolith-associated abdominal symptoms may be underestimated. This emphasizes the significance of taking appendicolith into account during the differential diagnosis of abdominal pain, particularly in instances where the clinical manifestations are atypical. Traditionally, management of an appendicolith in the absence of appendicitis has primarily involved observation, although some experts advocate for surgical removal of the appendix.^8^ The development of minimally invasive techniques, such as endoscopic retrograde appendicitis treatment (ERAT), offers a promising alternative to traditional surgical approaches, particularly in cases where appendicolith is present.

In recent years, ERAT has gained traction in the diagnosis and treatment of acute appendicitis, particularly within clinical practice across Chinese hospitals. By 2023, China had successfully performed over 10,000 ERAT procedures,^6^ and the technique has attracted growing attention as a minimally invasive alternative for appendiceal pathologies globally. Under the premise of preserving the appendix and its function, the appendix can be relieved from obstruction through ERAT procedure. The minimally invasive nature of ERAT means that patients typically experience less pain, shorter hospital stays, and a quicker return to normal activities. The procedure has shown promising results in clinical trials, with high success rates and low complication rates, making it an attractive option for patients and healthcare providers alike. Several subsequent randomized controlled trials have found that ERAT is effective in the treatment of acute symptomatic appendicitis.^9^^,^^10^^,^^11^^,^^12^^,^^13^^,^^14^ However, there have been no scientific studies or comprehensive reports on treatment of symptomatic appendicoliths.

## Results

### Baseline characteristics of patients

A total of 78 patients received ERAT for the treatment of appendiceal stones at Linyi people’s hospital between July 2022 and July 2023. No data on race or ethnicity was collected. These patients were categorized into two distinct groups based on their age: the adult group and the minor group.

The adult group had 47 patients,with an age range of 18–73 years and a median age of 41 years (IQR 21–58), and 59.6% were females. The median pre-ERAT Alvarado score was 2 (IQR 0–3). Forty-two patients (89.4%) had Alvarado score between 0 and 4, while only five patients (10.6%) had Alvarado score between 5 and 6. Thirty-six patients (76.6%) had abdominal pain as their main symptom and the rest had abdominal distension. The median pre-ERAT abdominal pain or distension questionnaire (APQ) score in the adult group was 6 (IQR 5–6) (Table 1).

**Table 1:** Baseline data: demography and symptom status

	Adults group (*n* = 47)	Minors group (*n* = 31)	*p* value
Age (year) median (IQR)	41 (21–58)	16 (14–17)	<0.001^a^
Gender(female), *n* (%)	28 (59.6)	15 (48.4)	0.344^b^
Alvarado score median (IQR)	2 (0–3)	2 (1–3)	0.942^a^
Alvarado score, *n* (%)			
0–4	42 (89.4)	28 (90.3)	0.939^b^
5–6	5 (10.6)	3 (9.7)	
APQ score median (IQR)	6 (5–6)	6 (5–6)	0.928^a^
Main symptom, *n* (%)			
Abdominal pain	36 (76.6)	23 (74.2)	0.895^b^
Abdominal discomfort	11 (23.4)	8 (25.8)	

a Mann-Whitney U test.

b Pearson’s Chi-squared test.

In the minor group, there were 31 patients,with an age range of 6–17 years and a median age of 16 years (IQR 14–17), and 48.4% were females. The median pre-ERAT Alvarado score was 2 (IQR 1–3). Twenty-eight patients (90.3%) had Alvarado score between 0 and 4, while only three patients (9.7%) had Alvarado score between 5 and 6. Twenty-three patients (74.2%) had abdominal pain as their main symptom and eight patients (25.8%) had abdominal distension. The median pre-ERAT APQ score in the minor group was 6 (IQR 5–6) (Table 1).

### Procedural details

The cannulation was successful in 46 patients (97.9%) in the adult group and all the patients in the minor group. In the adult group, there was a single instance where the insertion of the catheter encountered a failure because the anterior end of the appendix was twisted, which made it difficult to pass the catheter and incisional knife; but we successfully passed the guidewire to place a plastic stent. The stones were successfully removed in both groups.

During colonoscopy, the appendix orifice can be directly visualized. Iodinated contrast fluoroscopy allows direct measurement of the appendix lumen diameter. The presence of filling defects within the appendix lumen under fluoroscopy indicates irregular contours or potential obstructions. A measured diameter of less than 3 mm suggests luminal narrowing, whereas a diameter exceeding 5 mm indicates luminal dilation. If the appendiceal orifice exhibit significant edema or if luminal narrowing is observed, placement of a plastic stent is indicated to ensure patency and drainage.

Forty-four patients (89.8%) in the adult group and twenty-two patients (70.9%) in the minor group had varying degrees of congestion and swelling of the appendix opening. Purulent discharge oozing from the appendix opening occurred in 5 patients (10.2%) of the adult group and 4 patients (12.9%) of the minor group. Appendicoliths incarceration was seen in 2 patients (4.1%) of the adult group and 3 patients (9.7%) of the minor group. Iodine contrast fluoroscopy showed irregularity of the contour of the appendix in 26 adult patients (53.1%) and 9 minor patients (29.0%), narrowing of the appendix lumen in 30 adults (61.2%) and 11 minors (35.5%), and expansion of the appendix lumen in 9 adults (18.4%) and 7 minors (22.6%). The appendicolith morphology was mostly soft sediment-like fecal sludge (38 adults and 18 minors), and some fecal stones (9 adults and 13 minors). Most of the stones could be removed by saline flushing in 43 adult patients (91.5%) and 28 minor patients (90.3%). Some larger appendicoliths cannot be removed by saline irrigation alone and require retrieval with the aid of a mesh basket or balloon (4 adults and 3 minors). (Figure 1; Table 2). The plastic stent was placed in 33 patients (70.2%) in the adult group and 20 patients (64.5%) in the minor group (Table 2).

**Figure 1:**
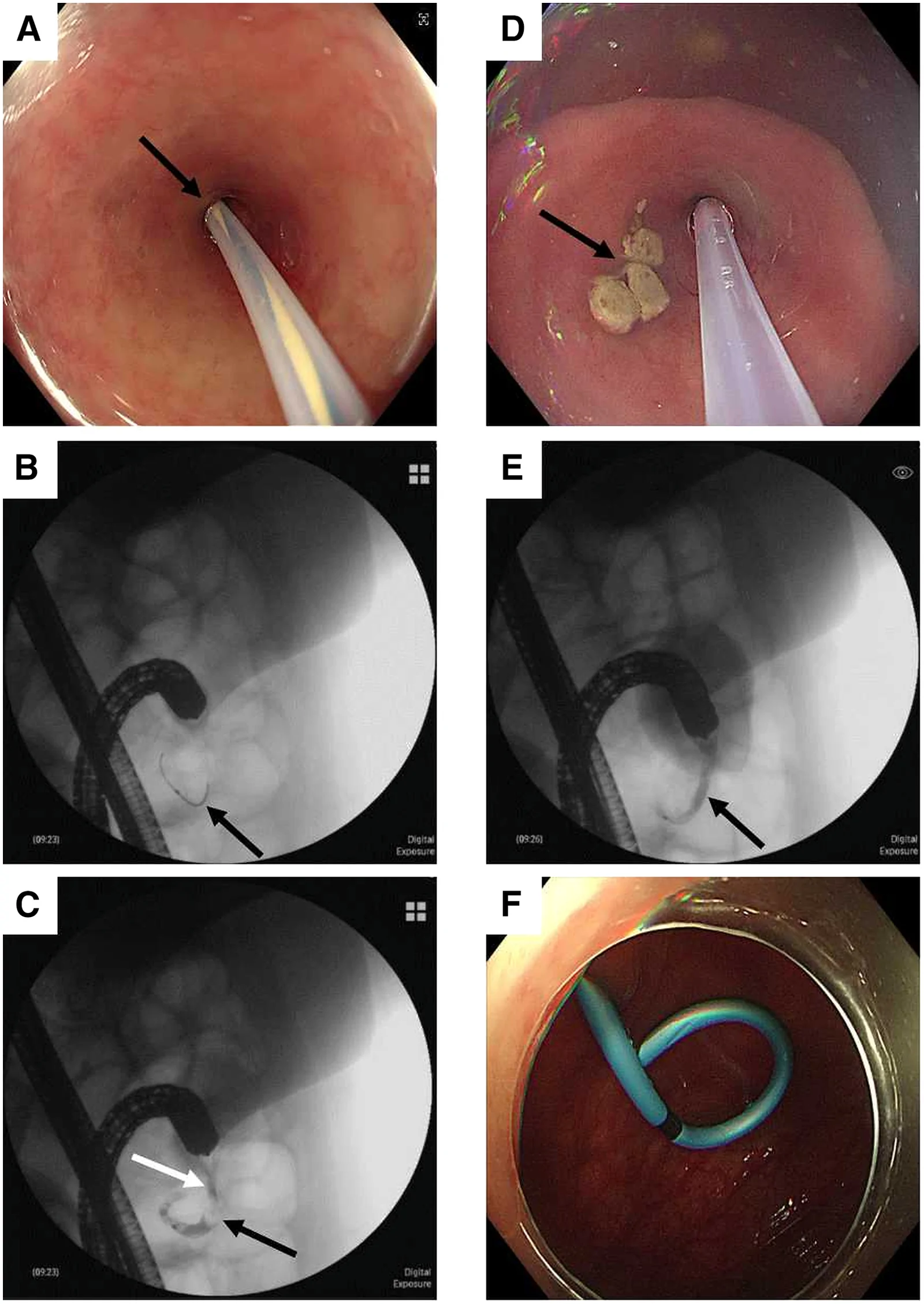
Procedure for ERAT in an adult patient (A) A contrast catheter is inserted through the opening of the appendix (black arrow). (B) Fluoroscopy shows insertion of the guidewire into the appendiceal lumen (black arrow). (C) Appendicography shows details of the appendiceal lumen, including the pre-treatment stenosis (white arrow) and filling defects (black arrow). (D) Flushing of fecal stones through the catheter, which is seen to flow out (black arrow). (E) Re-imaging of the appendiceal lumen is clear (black arrow), with no residual stone negative image. (F) Plastic stent placed.

**Table 2:** Procedural details

	Adults group (*n* = 47)	Minor group (*n* = 31)	*p* value
Operation, *n* (%)			
Cannulation	46 (97.9)	31 (100)	1.000^a^
Plastic stent placement	33 (70.2)	20 (64.5)	0.807^b^
Operation time (Min)	32.3 ± 12.4	30 (20–42)	0.851^c^
Image of the opening of the appendix, *n* (%)			
Congestion and swelling	44 (89.8)	22 (70.9)	0.469^a^
Purulent discharge	5 (10.2)	4 (12.9)	
Appendicoliths incarceration	2 (4.1)	3 (9.7)	
Fluoroscopic ERAT imaging,*n* (%)			
Irregular contour	26 (53.1)	9 (29.0)	0.404^a^
Lumen narrowing	30 (61.2)	11 (35.5)	
Lumen expansion	9 (18.4)	7 (22.6)	
Appendicoliths morphology, *n* (%)^d^			
Soft sludge	38 (80.9)	18 (58.1)	0.038^b^
Lumps of fecalith	9 (19.1)	13 (41.9)	
Method of stone removal, *n* (%)			
Saline flush	43 (91.5)	28 (90.3)	1.000^a^
Extracted by an extraction basket	1 (2.0)	1 (3.2)	
Extracted by a balloon catheter	3 (6.1)	2 (6.5)	
Number of days in hospital (day)	2 (2–3)	2 (2–3)	0.811^c^

Lumps of faecalith: appendicoliths that remain in a lump-like form after being flushed with physiological saline solution.

a Fisher’s Exact test.

b Pearson’s Chi-squared test.

c Mann-Whitney U test.

d Soft sludge: appendicoliths exhibited a sand-like appearance following irrigation with physiological saline solution.

### Short- and long-term symptomatic relief

The one-year post-ERAT follow-up period served as the study endpoint. To differentiate between short-term and long-term outcomes, a threshold of 6 months post-procedure was applied. Treatment response was assessed using the APQ score, with outcomes categorized as follows: complete remission (post-ERAT APQ score of 0), partial remission (post-ERAT APQ score lower than baseline), or no remission (post-ERAT APQ score equal to or greater than baseline).

In both patient cohorts, APQ scores predominantly clustered near zero throughout the recovery period. The proportion of patients achieving an APQ score of 0 demonstrated a consistent upward trend over time. In the adult group, this proportion inc0reased from 61.7% at one week to 74.5% at one month, 76.6% at six months, and 78.7% at one year post-ERAT. Similarly, in the minor group, the proportion rose from 71.0% at one week to 77.4% at one month, 83.9% at six months, and 87.1% at one year post-ERAT (Figures 2 and 3).

**Figure 2:**
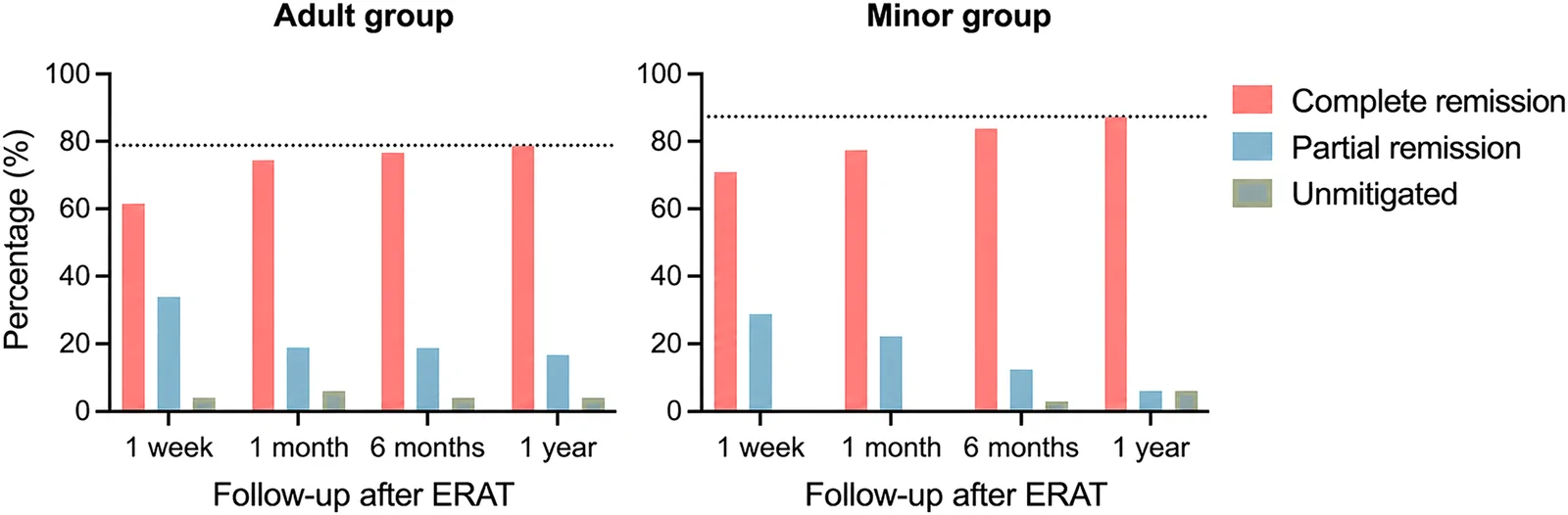
Short- and long-term symptomatic relief in adult and minor groups

**Figure 3:**
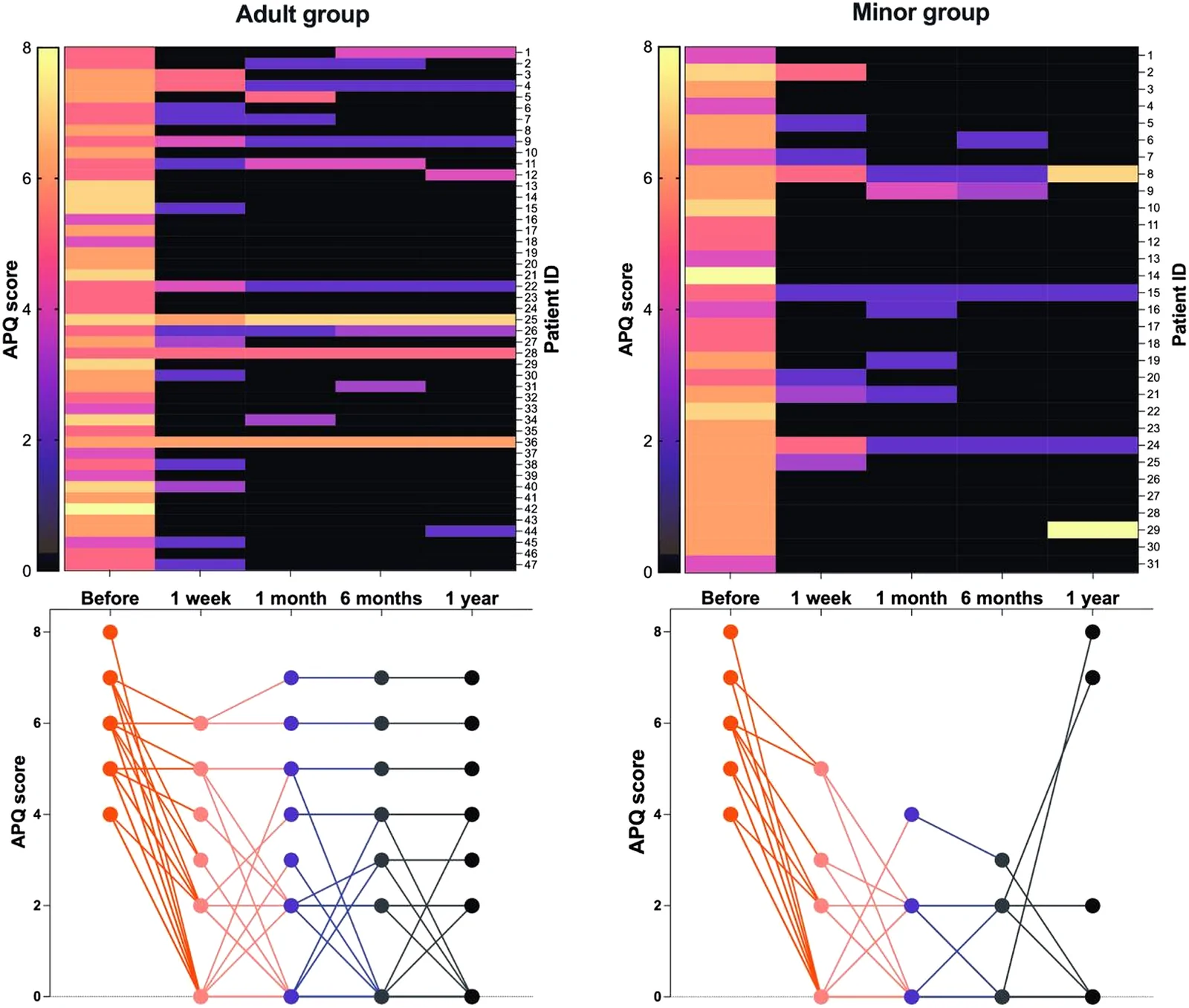
Distribution diagrams of APQ scores at different time intervals before and after ERAT in patients with appendicolith in the adult and minor groups

After 1-year follow-up, three patients in the adult group were unmitigated. Among the three patients, one patient had severe abdominal pain attributable to concurrent colonic diverticulitis; another presented recurrence of appendicoliths; and the third patient had developed acute appendicitis and had undergone surgical resection. In the minor group, two patients were unmitigated after 1-year follow-up. Among the two patients, one had recurrent appendicoliths and the other one developed acute appendicitis and underwent appendectomy. (Figure 2; Table 3).

**Table 3:** Symptom relief at 1 year after ERAT in the adult and minor groups

	Adults group (*n* = 47)	Minors group (*n* = 31)	*p* value
Complete remission *n* (%)	37 (78.7%)	27 (87.0%)	0.548^a^
Partial remission *n* (%)	7 (14.9%)	2 (6.5%)	0.470^a^
Unmitigated *n* (%)	3 (6.4%)	2 (6.5%)	1.000^b^

a Pearson’s Chi-squared test.

b Fisher’s Exact test.

### Cox regression analysis

Table 4 and Table S1 shows univariate and multivariate analyses of factors independently predictive of incomplete remission after ERAT. In the adult group, disease duration >3 months (multivariate hazard ratio 5.3; 95% CI, 1.3–22), NLR >1.87 (multivariate hazard ratio 0.17; 95% CI, 0.034–0.85), and appendicolith morphology in the form of lumps (multivariate hazard ratio 4.8; 95% CI, 1.2–18) remained statistically significant. Because the endpoint was incomplete remission, The factors found to be predictive in the final results in the adult group were disease duration >3 months and appendicolith morphology in the form of lumps,the HR below 1 for NLR >1.87 indicates an inverse association with incomplete remission rather than an increased risk. No significant independent factors were identified in the minor group.

**Table 4:** Analyzing risk factors for incomplete remission after ERAT in the adult group using Cox proportional risk models

Adult group	Univariate		Multivariate	
Variables	Hazard ratio (95% cl)	*p* Value	Hazard ratio (95% cl)	*p* Value
Male gender	0.7 (0.22–2.2)	0.54	NA	NS
Age≥41year	0.78 (0.25–2.4)	0.67	NA	NS
Alvarado score	3.3 (0.42–26)	0.26	–	–
Abdominal pain	1.1 (0.8–1.5)	0.55	NA	NS
APQ≥6	0.79 (0.25–2.5)	0.69	NA	NS
Duration > 3 months	4 (1.1–14)	0.035^a^	5.3 (1.3–22)	0.022^a^
BMI > 24	0.38 (0.1–1.4)	0.14	NA	NS
BMI<18.5	1.7 (0.44–6.3)	0.45	NA	NS
NLR > 1.87	0.13 (0.027–0.65)	0.013^a^	0.17 (0.034–0.85)	0.031^a^
PLR > 129.41	1.3 (0.39–4.1)	0.7	NA	NS
Not only saline flush	1.3 (0.16–10)	0.83	NA	NS
Stent	0.67 (0.19–2.3)	0.52	NA	NS
Lumps of appendicolith	3.6 (1.1–12)	0.035^a^	4.8 (1.2–18)	0.024^a^
Surgical duration > 30m	0.33 (0.079–1.4)	0.12	NA	NS
Hospitalization>2days	0.43 (0.094–2)	0.28	NA	NS

NA, not assessed; NS, not significant; BMI, body mass index; NLR, neutrophil-lymphocyte ratio; PLR, platelet-lymphocyte ratio. (Both are indicators of inflammatory and immune status in blood tests).

a *P* < 0.05 (two-sided).

## Discussion

Appendicoliths are considered a significant predisposing factor for appendicitis, and when appendicitis is accompanied by obstruction of appendicoliths, it is concurrently associated with a substantially heightened risk of severe complications, including perforation, abscess, and gangrene.^15^^,^^16^ Most patients with appendicoliths are asymptomatic, and some are found incidentally when checking for various abdominal discomforts. There is no standard treatment plan for symptomatic appendicoliths. Traditionally, some surgeons recommend that appendectomy should be considered once appendicoliths are diagnosed to be present.^2^^,^^17^ However, the decision to perform an appendectomy is not always straightforward. The presence of an appendicolith does not necessarily mean that the appendix is currently inflamed or at immediate risk of rupture. Some clinicians advocated conservative antibiotic treatment for acute uncomplicated appendicitis. Studies have indicated that there is no significant difference in treatment success rates between medical management and ERAT.^18^ However, the former carries a higher overall recurrence rate, particularly in patients with appendicoliths, who experience recurrence at a shorter interval.^1^ Compared to patients without an appendicolith, these with one are at a higher risk of progressing to complicated appendicitis (e.g., perforation, gangrene, or abscess),^7^^,^^19^ often necessitating appendectomy. Further research is needed to establish clear guidelines for the management of symptomatic appendicoliths, which could improve patient outcomes and avoid unnecessary surgeries.

ERAT is established for managing acute simple appendicitis,^9^^,^^11^^,^^20^ yet its use for symptomatic appendicoliths remains unreported. The adaptation of ERAT for the treatment of symptomatic appendicoliths is a promising prospect due to its minimal invasiveness and the potential to mitigate the risks linked with conventional surgical techniques. This is the first retrospective study with a sample size of 78 cases to investigate the association between appendicoliths and abdominal symptoms and to evaluate the efficacy and safety of the ERAT procedure in this specific patient population.

The endoscopic and fluoroscopic findings in this cohort suggest a plausible link between appendicoliths and chronic abdominal symptoms. The results indicated that 42 patients (89.4%) in the adult group and 22 patients (70.9%) in the minor group had varying degrees of congestion and swelling of the appendiceal opening, indicating that appendicoliths may induce localized mechanical obstruction and low-grade inflammation, which could manifest clinically as recurrent pain or distension. This hypothesis is further supported by the predominance of soft, sludge-like appendicoliths (38 adults and 18 minors), which likely contribute to intermittent symptoms rather than the acute obstruction typical of hard fecaliths. Iodine contrast fluoroscopy showed irregularity of the contour of the appendix in 26 adult patients (53.1%) and 9 minor patients (29.0%), narrowing of the appendiceal lumen in 30 adults (61.2%) and 11 minors (35.5%), and expansion of the appendiceal lumen in 9 adults (18.4%) and 7 minors (22.6%). Most of the stones could be removed by saline flushing in 43 adult patients (91.5%) and 28 minor patients (90.3%). This high technical success rate of saline flushing during ERAT aligns with this morphological characteristic and provides a direct therapeutic rationale for endoscopic intervention.

The long-standing symptoms and anatomical findings in this cohort also raise the possibility that some cases overlapped with subacute or chronic appendicitis rather than representing purely mechanical symptomatic appendicoliths. Chronic appendicitis has been described as persistent or recurrent, usually less severe abdominal pain lasting weeks to months, often related to partial or intermittent luminal obstruction; reported causes include fecaliths, and imaging findings may resemble acute appendicitis, including appendicoliths and luminal or periappendiceal inflammatory changes.^21^^,^^22^ Because histopathology was unavailable after ERAT and patients with Alvarado score ≥7 or obvious systemic infection were excluded, we cannot definitively classify these patients as chronic appendicitis. We herefore interpret symptomaappendicoliths as a clinical phenotype that may include low-grade, subacute, or chronic appendiceal inflammation in a subset of patients.

ERAT demonstrated significant efficacy in achieving symptomatic relief for patients with appendicoliths. In adults, the rate of complete remission (APQ = 0) increased from 61.7% at one week post-procedure to 74.5% at one month and 76.6% at six months. A more pronounced trend was observed in minors, with remission rates rising from 71.0% at one week to 77.4% at one month and 83.9% at six months. At one-year follow-up, over 93% of patients in both cohorts reported meaningful symptomatic improvement, with complete remission achieved in 78.7% of adults and 87.1% of minors. Prognostic analysis identified disease duration exceeding three months (HR 5.3; 95% CI, 1.3–22) and lumps of appendicolith (HR 4.8; 95% CI, 1.2–18) as factors associated with incomplete remission in adults. By contrast, NLR >1.87 showed an HR below 1 (HR 0.17; 95% CI, 0.034–0.85), indicating an inverse association with incomplete remission rather than a risk factor. This unexpected finding should be interpreted with caution. An elevated NLR typically indicates the presence of a local, low-grade inflammatory response in the body and can effectively reflect the inflammatory changes induced by appendicoliths. Our analysis suggests that a mildly elevated NLR may exhibit a protective effect, which may be related to the presence of a mild, localized and controllable inflammatory response in the patients appendix; A moderate inflammatory response may promote the absorption of local exudate and reduce luminal edema following stone removal, thereby facilitating symptom improvement. Based on this characteristic, patients of this type tend to achieve better treatment outcomes with ERAT, with more significant postoperative relief of clinical symptoms. Conversely, patients with an NLR ≤1.87 may have an insufficient inflammatory response and impaired local drainage, leading to persistent symptoms and a lack of significant relief. However, one cannot entirely rule out residual confounding factors, a small sample size within the subgroup, or heterogeneity in baseline inflammation levels; it is not certain that a higher NLR confers a causal protective effect. The above factors indicate that the severity of chronicity, the presence of systemic inflammation, and appendicolith characteristics collectively influence treatment outcomes.

Although no external patient-level cohort of ERAT for symptomatic appendicoliths is currently available, we compared our findings with published datasets on ERAT for uncomplicated appendicitis and appendicolith-associated appendicitis. In prospective ERAT cohorts for uncomplicated acute appendicitis, reported technical and clinical outcomes have been favorable,^9^^,^^20^ which is broadly consistent with our high cannulation rate, complete stone removal, and over 93% meaningful symptomatic improvement at 1 year. By contrast, antibiotic-based management of appendicitis in patients with appendicoliths has been associated with a higher risk of complicated appendicitis and subsequent appendectomy in large randomized data.^19^ This external comparison supports the biological plausibility of appendicolith removal as an organ-preserving approach, while also underscoring that our results require independent validation in multicenter cohorts.

The strength of our study lies in its pioneering evaluation of ERAT for symptomatic appendicoliths in both adult and minor patients. Our findings substantiate that ERAT offers considerable potential for achieving sustained symptomatic relief. The marked and durable resolution of symptoms following appendicolith removal supports the conclusion that abdominal pain or distension in these patients were mainly associated appendicoliths. We consider this treatment regimen to be safe and effective for individuals across all age groups who can take colonoscopy, supporting its recommendation as a viable therapeutic strategy for managing symptomatic appendicoliths.

### Limitations of the study

This single-center, retrospective study is inherently limited by its small sample size, potential selection bias, and challenges in controlling confounders, which may affect generalizability. In addition, symptom improvement was based on patient-reported APQ scores from patients within one year after ERAT and the study lacked a sham endoscopic observation control or randomized comparator; therefore, placebo effects, regression to the mean, and spontaneous symptom resolution cannot be fully excluded.^23^ Future multicenter, prospective studies with external validation cohorts, randomized comparators and, where ethically feasible, sham-controlled designs are needed to validate these findings.

## Resource availability

### Lead contact

Further information and requests for resources should be directed to and will be fulfilled by the lead contact, Chao Du (duchao2021@hotmail.com).

### Materials availability

This study did not generate new unique reagents.

### Data and code availability

• Access to the patient-level data are restricted under hospital policy and the study’s Institutional Review Board approval because public release could compromise patient confidentiality. Requests for access should be directed to the lead contact and will be considered subject to institutional and ethical approval.
• This article does not report original code.
• Any additional information required to reanalyze the data reported in this article is available from the lead contact upon request.

## Acknowledgments

This work was supported by 10.13039/501100007129Shandong Province Natural Science Foundation (no. ZR2021MH183) and Shandong Province Medical and Health Science and Technology Project (202503030109).

## Author contributions

Conception and design, C.D., L.K., and L.L.; analysis and Interpretation of the data, R.L., A.W., X.Z., X.X., Q.Z., T.J., and J.N.; drafting of the article, R.L., L.K., and C.D.; critical revision of the article for important intellectual content, R.L., L.K., and C.D.; final approval of the article, R.L., A.W., X.Z., X.X., Q.Z., T.J., J.N., L.L., L.K., and C.D.

## Declaration of interests

The authors declare that they have no conflict of interest.

## STAR★Methods

### Key resources table

**Table:** 

REAGENT or RESOURCE	SOURCE	IDENTIFIER
**Biological samples**		

Human participants with symptomatic appendicoliths (adults, *n* = 47; minors, *n* = 31)	Linyi People’s Hospital; July 2022–July 2023	N/A

**Deposited data**		

Patient-level clinical data underlying this study	This paper; available from the lead contact upon request, subject to institutional and ethical approval	N/A

**Experimental models: Organisms/strains**		

Human: patients undergoing endoscopic retrograde appendicitis therapy (ERAT) for symptomatic appendicoliths	Linyi People’s Hospital	N/A

**Software and algorithms**		

IBM SPSS Statistics, version 25.0	IBM Corp., Armonk, NY, USA	https://www.ibm.com/products/spss-statistics
GraphPad Prism, version 10	GraphPad Software, Boston, MA, USA	https://www.graphpad.com/features

**Other**		

Colonoscope, contrast catheter, guidewire, iodinated contrast fluoroscopy, incisional knife, irrigation, and plastic appendiceal stent used for ERAT	Linyi People’s Hospital	N/A

### Experimental model and study participant details

Human participants. This single-center retrospective study included 78 patients who underwent ERAT for symptomatic appendicoliths at Linyi People’s Hospital between July 2022 and July 2023. The study has been approved by the Linyi People’s hospital’s ethics committee, and the ethics approval number is YY200607. Participants were allocated by age to an adult group (*n* = 47; age 18–73 years; 28 female and 19 male) or a minor group (*n* = 31; age 6–17 years; 15 female and 16 male). Appendicoliths were identified by abdominal ultrasonography or computed tomography. We used the Alvarado score to rule out acute appendicitis and the APQ score to assess patients’ abdominal symptoms. The scoring standard for acute appendicitis and abdominal symptoms is shown in table below.^24^^,^^25^^,^^26^ Eligible participants had an Alvarado score <7, no marked elevation of infection markers, and an APQ score ≥4. Exclusion criteria were absence of informed consent; known or suspected cancer or infectious disease; another known cause of abdominal pain, including a functional gastrointestinal disorder meeting Rome IV criteria; or pregnancy. Sex was included in the prognostic analyses, and no statistically significant association between sex and incomplete remission was identified; sex-specific inference remains limited by the sample size and retrospective design.

**Table:** Scoring standard

Alvarado score standard		
	1	2
Metastatic right lower abdominal pain	**✓**	–
Poor appetite	**✓**	–
Nausea and vomiting	**✓**	–
Right lower abdominal tenderness	–	**✓**
Rebound tenderness	**✓**	–
Fever ≥37.5°C	**✓**	–
Leukocytosis	–	**✓**
Neutrophil percentage↑	**✓**	–
∗0–4, Appendicitis is highly unlikely 5–6, Appendicitis is possible ≥7, appendicitis very likely		

Abdominal pain/distension questionnaire (APQ score)^a^		
Score	Symptom	Frequency
0	None	None
1	Minor (not affecting daily activities)	1 day per week
2	Relevant (affects activities, but not enough to change them)	2–3 days per week
3	Severe (significantly affects activities enough to change them)	4–6 days per week
4	Extremely severe (interference with daily activities)	Everyday

a Symptom scores and frequency scores are added together for a total score.

### Method details

#### ERAT procedure

The patient’s intestine is cleaned with magnesium sulfate and simethicone, and after intravenous anesthesia, a conical transparent cap is placed on the anterior end of the colonoscopy, and a single hook and pull method is used to enter the lens through the lumen to the ileocecal region to find the opening of the appendix, and the transparent cap is used to open the Gerlach’s flap to fully expose the opening of the appendix. If the appendiceal opening is detected, a catheter can be inserted under the guidance of a guide-wire. After successful insertion of the catheter, the contrast agent is injected, the appendix is visualised, the guide-wire is withdrawn, and the appendiceal lumen is observed for intra-appendiceal contrast. If a filling defect is found, the appendiceal lumen can be flushed with saline to flush out the appendicolith, and the balloon or mesh basket can be used to remove the stone if necessary. After the stone is removed and the flushing solution is clarified, the operation is finished with no stone remaining in the appendiceal lumen. If the appendix opening is significantly swollen or the lumen is narrowed, a stent may be placed within the appendix cavity to facilitate drainage.

#### Follow-up and outcome assessment

All patients were followed up for 1 year after ERAT in adult and minor patients. The main outcome was short- and long-term symptom relief by employing a six-month postoperative marker as the dividing threshold. The patients were considered complete remission if the post-ERAT APQ score was 0, partial remission if the post-ERAT APQ score was lower than that of the pre-ERAT, and no remission if the post-ERAT APQ score was greater than or equal to that of the pre-ERAT APQ score.

The secondary outcomes included an analysis of the influence of gender, age, the APQ score, Body Mass Index (BMI), disease duration, blood counts and appendicolith morphology on long-term symptomatic relief. By considering these multiple variables, we aimed to gain a more nuanced understanding of the factors that contribute to sustained symptomatic relief following the surgical intervention.

### Quantification and statistical analysis

Categorical variables are reported as counts and percentages. Continuous variables are reported as medians with interquartile ranges (IQRs), except operation time where the table reports mean ± SD. Between-group comparisons used the Mann-Whitney U test for continuous variables and Pearson’s chi-square test or Fisher’s exact test for categorical variables, as specified in the table footnotes. The exact sample size was 78 participants (47 adults and 31 minors), and n represents individual participants. Univariate Cox proportional hazards models evaluated factors associated with incomplete remission; variables with *p* < 0.05 were entered into the multivariable Cox model, and *p* < 0.05 in the multivariable model was considered statistically significant. Hazard ratios are reported with 95% confidence intervals. Statistical details are provided in the Results and Tables 2, 3, 4, and 5. All statistical analyses were performed using IBM SPSS Statistics, version 25.0 (IBM Corp., Armonk, NY, USA). Figures were generated using GraphPad Prism, version 10 (GraphPad Software, Boston, MA, USA).
