# Supplementary material for: One year follow up outcomes of endoscopic retrograde appendicitis treatment for symptomatic appendicoliths
Source: iScience. 2026 Sep 24;29(10):117571. doi: 10.1016/j.isci.2026.117571 (PMC13634889; doi:10.1016/j.isci.2026.117571)
Supplement: Supplementary file 1 [file mmc1.pdf]

iScience, Volume *29*

Supplemental information

One year follow up outcomes of endoscopic retrograde appendicitis treatment for symptomatic appendicoliths

Ran Li, Aihua Wang, Xiutian Zhang, Xiangming Xu, Qingqing Zhang, Tao Ji, Jiao Nie, Lin Lu, Lingjian Kong, and Chao Du

Figure S1: Endoscopic retrograde appendicitis treatment in a minor patient.

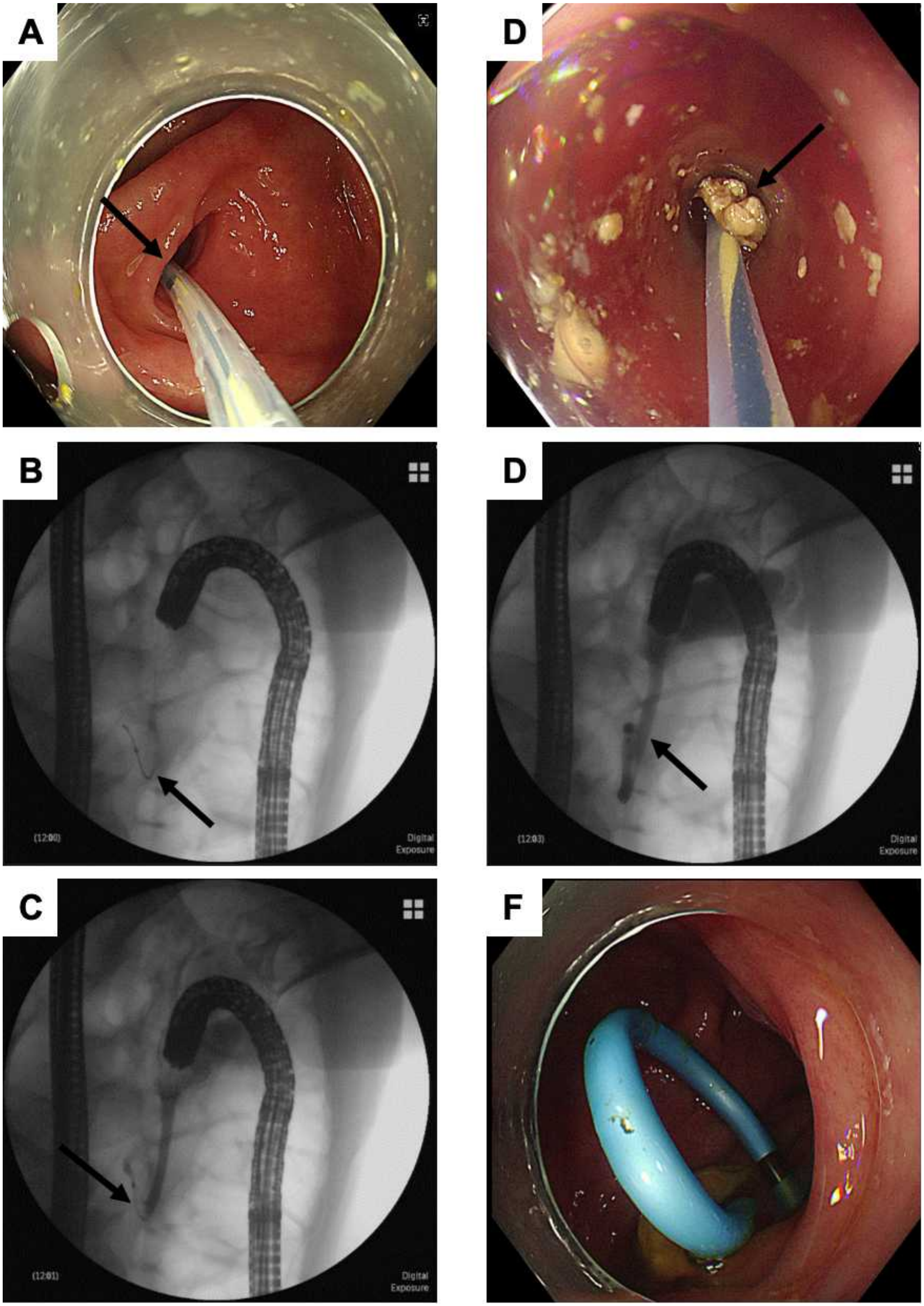

A, A contrast catheter is inserted through the appendiceal orifice (black arrow). **B,** Fluoroscopy shows insertion of the guidewire into the appendiceal lumen (black arrow). **C,** Appendicography shows the appendiceal lumen and filling defects (black arrow). **D,** Fecaliths are flushed through the catheter (black arrow). **E,** Repeat appendicography shows a clear lumen without residual filling defects (black arrow). **F,** A plastic stent is placed.

Table S1: Cox proportional hazards analysis of factors associated with incomplete remission after ERAT in the minor group.

Minor group	Univariate	Univariate	Multivariate	Multivariate
**Variables**	**Hazard ratio (95% CI)**	**P value**	**Hazard ratio (95% CI)**	**P value**
Male gender	0.65 (0.11-3.9)	0.64	NA	NS
Age≥16y	0.48 (0.067-3.5)	0.47	NA	NS
Abdominal pain	3.5e+08 (0-Inf)	1	NA	NS
Alvarado score	1 (0.48-2.1)	0.99	NA	NS
APQ≥6	1.2 (0.12-12)	0.86	NA	NS
Duration>3 months	0.3 (0.033-2.7)	0.28	NA	NS
BMI>24	1.7 (0.24-12)	0.6	NA	NS
BMI<18.5	1.1e+13 (0-Inf)	1	NA	NS
NLR>1.60	0.58 (0.077-4.4)	0.6	NA	NS
PLR>120.42	1.1 (0.15-7.6)	0.95	NA	NS
Not only saline flush	4.4 (0.39-48)	0.23	NA	NS
Stent	1.2e+09 (0-Inf)	1	NA	NS
Lumps of appendicolith	3.6 (0.37-35)	0.27	NA	NS
Surgical duration>30m	0.92 (0.13-6.7)	0.94	NA	NS
Hospitalization>2d	1.2 (0.2-7.4)	0.84	NA	NS

NA, not assessed; NS, not significant; BMI, body mass index; NLR, neutrophil-to-lymphocyte ratio; PLR, platelet-to-lymphocyte ratio. Hazard ratios are shown with 95% confidence intervals.
